# Supplementary material for: Integration of ATAC- and RNA-sequencing identifies chromatin and transcriptomic signatures in classical and non-classical zebrafish osteoblasts and indicates mechanisms of entpd5a regulation
Source: eLife. 2026 Oct 27;13:RP100230. doi: 10.7554/eLife.100230 (PMC13713029; doi:10.7554/eLife.100230)
Supplement: Supplementary file 2 [file elife-100230-supp1.docx]

| Sample | FLDD | TSS score | Sample | FLDD | TSS score |
| --- | --- | --- | --- | --- | --- |
| Osteoblasts rep1 | 0,267 | 2,332 | ***entpd5a*+ NSCs rep1** | 0,259 | 2,168 |
| Osteoblasts rep2 | 0,207 | 4,996 | ***entpd5a*+ NSCs rep2** | 0,258 | 2,479 |
| Osteoblasts rep3 | 0,219 | 4,236 | ***entpd5a*+ NSCs rep3** | 0,231 | 4,245 |
| Chondrocytes rep1 | 0,211 | 6,177 | ***entpd5a*- NSCs rep1** | 0,299 | 3,511 |
| Chondrocytes rep2 | 0,215 | 4,244 | ***entpd5a*- NSCs rep2** | 0,182 | 4,507 |
| Chondrocytes rep3 | 0,230 | 3,020 | ***entpd5a*- NSCs rep3** | 0,181 | 2,097 |
