# Supplementary material for: Integration of ATAC- and RNA-sequencing identifies chromatin and transcriptomic signatures in classical and non-classical zebrafish osteoblasts and indicates mechanisms of entpd5a regulation
Source: eLife. 2026 Oct 27;13:RP100230. doi: 10.7554/eLife.100230 (PMC13713029; doi:10.7554/eLife.100230)
Supplement: Supplementary file 3 [file elife-100230-supp2.docx]

| Construct | Primer 1 (5’ 🡪 3’) | Primer 2 (5’ 🡪 3’) |
| --- | --- | --- |
| *entpd5a(2.2):GFP* | AACCGGAATTCCATGAATGTACAGACTGCC | TACCGGAATTCTTTCTCCCACGCCG |
| *entpd5a(5.7):GFP – NEBuilder fragment 1* | GTTTCCTACCGGGTGCTCCGGTTTCCCT | CCGTGCCATAGCAAAAGTGGATAGTGCATCTATGAGCTAATTCAGTAGGTG |
| *entpd5a(5.7):GFP – NEBuilder fragment 2* | GGATCCACTAAGCCACCGTGCCGCGGATCCACTAAGCCACCGTGCCGC | CGGAGCACCCGGTAGGAAACCCAAACGAACTCG |
| *entpd5a(5.7):GFP 1.4kb fragment* | CGCGGATCCACATCGCACAGTGAGA | CGCGGATCCACAGTCTACATGAATGC |
| *entpd5a(2.2-introns):GFP* | [Phos]GCTATACTACCTTCATTGATGCC | [Phos]ACCCAGGTAAATCTTTTAATGTCC |
| *entpd5a(2.2-coq6):GFP* | [Phos]GGAACTGCTTCTAATGCTGC | [Phos]AACAAAGCATTAGAGATCCAGC |
| *entpd5a(del243):GFP* | [Phos]CTTGTCAGAAGTCAATATGAGGGC | [Phos]GATCTATTTAAATTAAACTGGGC |
| *entpd5a(d418):GFP* | [Phos]CAAACTCCACCCAGGTCC | [Phos]GATCTATTTAAATTAAACTGGGC |
| *entpd5a(d665):GFP* | [Phos]GTTGTCGTAATGCAATCCTGC | [Phos]GATCTATTTAAATTAAACTGGGC |
| *entpd5a(d880):GFP* | [Phos]CCAATGAAAAGGTATTCACTTGC | [Phos]GATCTATTTAAATTAAACTGGGC |
| *entpd5a(r37):GFP* | [Phos]GGAAAGAAAGTGTAATATTTTTAGTTCC | [Phos]GGTCTTTTCTACCGTGCCAAGC |
| *entpd5a(r31):GFP* | [Phos]TTAAGAATGGCTCTTGATGC | [Phos]GCAAGTGAAAGAGAAAAAGAC |
