# Supplementary material for: Integration of ATAC- and RNA-sequencing identifies chromatin and transcriptomic signatures in classical and non-classical zebrafish osteoblasts and indicates mechanisms of entpd5a regulation
Source: eLife. 2026 Oct 27;13:RP100230. doi: 10.7554/eLife.100230 (PMC13713029; doi:10.7554/eLife.100230)
Supplement: Peer review history

# Editor report: eLife Assessment

**DOI:** 10.7554/eLife.100230.3.sa0

**Authors:** Koichi Kawakami

**Affiliations:** National Institute of Genetics Japan

**Keywords:** Compelling, Convincing, Fundamental

This **fundamental** work describes for the first time the combined gene expression and chromatin structure at the genome level in isolated chondrocytes and classical (cranial) and non-classical (notochordal) osteoblasts. In a **compelling** analysis of RNA-Seq and ATAC data, the authors characterize the two osteoblast populations relative to their associated chondrocyte cells and further proceed with a **convincing** analysis of the crucial entpd5a gene regulatory elements by investigating their respective transcriptional activity and specificity in developing zebrafish.

# Referee report: Reviewer #1 (Public review)

**DOI:** 10.7554/eLife.100230.3.sa1

Summary:

This work uses transgenic reporter lines to isolate entpd5a+ cells representing classical osteoblasts in the head and non-classical (osterix-) notochordal sheath cells. The authors also include entpd5a- cells, col2a1a+ cells to represent the closely associated cartilage cells. In a combination of ATAC and RNA-Seq analysis, the genome-wide transcriptomic and chromatin status of each cell population is characterized, validating their methodology and providing fundamental insights into the nature of each cell type, especially the less well-studied notochordal sheath cells. Using these data, the authors then turn to a thorough, and convincing analysis of the regulatory regions that control the expression of the entpd5a gene in each cell population. Determination of transcriptional activities in developing zebrafish, again combined with ATAC data and expression data of putative regulators results in a compelling, and detailed picture of the regulatory mechanisms governing expression of this crucial gene.

Strengths:

The major strength of this paper is the clever combination of RNA-Seq and ATAC analysis, further combined with functional transcriptional analysis of the regulatory elements of one crucial gene. This results in a very compelling story.

Weaknesses:

No major weakness, except for all the follow-up experiments that one can think of, but that would be outside of the scope of this paper.

Comments on revisions:

The description of Supplementary Figure 1 is still confusing: in the results section, it says "We photo converted and directly imaged entpd5a:Kaede positive embryos starting from the 15 somite- stage (s), when we could first detect the fluorophore along the newly-formed notochord progenitor cells (Suppl. Fig. 1E). We repeated photoconversion and imaging at 18, 21 and 24s (Suppl. Fig. 1F-H). ...(Suppl. Fig 1E)"

In the response, the authors say "we could see new Kaede expression under the control of the entpd5a promoter region within 1.5 hours of photoconversion, as shown in Suppl. Figure 1E-H."

In the legend to Suppl. Fig. 1, it says "Using the entpd5a:Kaede photoconversion line we first detect entpd5a expression at the 15 somite-stage (E). Following the same embryo, active expression of the gene continues until prior to 24 hpf (F-H)."

So my questions are: -was there a delay between photoconversion and imaging - was the same delay used for all pictures - was there indeed additional photoconversion for Fig.1 F-H before imaging?

This could be stated in Materials and Methods, and maybe in the legend to Suppl. Fig. 1

All other issues have been addressed.

# Referee report: Reviewer #2 (Public review)

**DOI:** 10.7554/eLife.100230.3.sa2

Summary:

Complementary to mammalian models, zebrafish has emerged as a powerful system to study vertebrate development and serve as a go-to model for many human disorders. All vertebrates share the ancestral capacity to form a skeleton. Teleost fish models have been a key model to understand the foundations of skeletal development and plasticity, pairing with more classical work in amniotes such as the chicken and mouse. However, the genetic foundation of the diversity of skeletal programs in teleosts have been hampered by mapping similarities from amniotes back and not objectively establishing more ancestral states. This is most obvious in systematic, objective analysis of transcriptional regulation and tissue specification in differentiated skeletal tissues. Thus, the molecular events regulating bone-producing cells in teleosts have remained largely elusive. In this study, Petratou et al. leverage spatial experimental delineation of specific skeletal tissues -- that they term 'classical' vs 'non-classical' osteoblasts -- with associated cartilage of the endo/peri-chondrial skeleton and inter-segmental regions of the forming spine during development of the zebrafish, to delineate molecular specification of these cells by current chromatin and transcriptome analysis. The authors further show functional evidence of the utility of these datasets to identify functional enhancer regions delineating entp5 expression delineated in 'classical' or 'non-classical' osteoblast populations. By integration with paired RNA-seq, they delineate broad patterns of transcriptional regulation of these populations as well as specific detail of regional regulation via predictive binding sites within ATACseq profiles. Overall the paper was very well written and provides an essential contribution to the field that will provide a foundation to promote modeling of skeletal development and disease in an evolutionary and developmentally informed manner.

Strengths:

Taken together, this study provides a comprehensive resource of ATAC-seq and RNA-seq data that will be very useful for a wide variety of researchers studying skeletal development and bone pathologies. The authors show specificity in the different skeletal lineages and show utility of the broad datasets for defining regulatory control of gene regulation in these different lineages, providing the foundation for hypothesis testing of not only agents of skeletal change in evolution but also function of genes and variations of unknown significance as it pertains to disease modeling in zebrafish. The paper is excellently written, integrating a complex history and experimental analysis into a useful and coherent whole. The terminology of 'classical' and 'non-classical' will be useful for the community in discussing biology of skeletal lineages and their regulation.

Weaknesses:

Two items arose that proposed areas for extending the description to integrate the data into the existing data on role of non-classical osteobasts and establishment/canalization of this lineage of skeletal cells.

(1) It was unclear how specific the authors' experimental dissection of head/trunk was in isolating different entp5a osteoblast populations. Obviously, this was successful given the specificity in DEG of results, however an analysis of contaminating cells/lineages in each population would be useful - e.g. maybe use specific marker genes to assess. The text uses terms such as 'specific to' and 'enriched in' without seemingly grounded meaning of the accuracy of these comments. Is it really specific e.g. not seen in one or other dataset, or is there some experimental variation in this?

(2) Further, it would be valuable to discuss NSC-specific genes such as calymmin (Peskin 2020) which has species and lineage specific regulation of non-classical osteoblasts likely being a key mechanistic node for ratcheting centra-specific patterning of the spine in teleost fishes. What are dynamics observed in this gene in datasets between the different populations, especially when compared with paralogues - is there obvious cis-regulatory changes that correlate with the co-option of this gene in early regulation of non-classical osteoblasts? The addition of this analysis/discussion would anchor discussions of a differential between different osteoblasts lineages in the paper.

Comments on revisions: All issues have been addressed.

# Referee report: Reviewer #3 (Public review)

**DOI:** 10.7554/eLife.100230.3.sa3

Summary:

This study characterizes classical and nonclassical osteoblasts as both types were analyzed independently (integrated ATAC-seq and RNAseq). It was found that gene expression in classical and nonclassical osteoblasts is not regulated in the same way. In classical osteoblasts Dlx family factors seem to play an important role, while Hox family factors are involved in the regulation of spinal ossification by nonclassical osteoblasts. In the second part of the study, the authors focus on the promoter structure of entpd5a. Through the identification of enhancers they reveal complex modes of regulation of the gene. The authors suggest candidate transcription factors that likely act on the identified enhancer elements. All the results taken together provide comprehensive new insights into the process of bone development, and point to spatio-temporally regulated promoter/enhancer interactions taking place at the entpd5a locus.

Strengths:

The authors have succeeded in justifying a sound and consistent buildup of their experiments, and meaningfully integrate the results into the design of each of their follow-up experiments. The data are solid, insightfully presented, and the conclusion valid. This makes this manuscript of great value and interest to those studying (fundamental) skeletal biology.

Weaknesses:

The study is solidly constructed, the manuscript is clearly written and the discussion is meaningful - I see no real weaknesses.

# Author comment: Author response

**DOI:** 10.7554/eLife.100230.3.sa4

**Authors:** Kleio Petratou, Martin Stehling, Ferenc Müller, Stefan Schulte-Merker

**Affiliations:** University of Münster Münster Germany; Max Planck Institute for Molecular Biomedicine Münster Germany; University of Birmingham Birmingham United Kingdom; WWU Muenster Muenster Germany

The following is the authors’ response to the original reviews.

**Public Reviews:**

**Reviewer #1 (Public Review):**

Summary:

This work uses transgenic reporter lines to isolate entpd5a+ cells representing classical osteoblasts in the head and non-classical (osterix-) notochordal sheath cells. The authors also include entpd5a- cells, col2a1a+ cells to represent the closely associated cartilage cells. In a combination of ATAC and RNA-Seq analysis, the genome-wide transcriptomic and chromatin status of each cell population is characterized, validating their methodology and providing fundamental insights into the nature of each cell type, especially the less well-studied notochordal sheath cells. Using these data, the authors then turn to a thorough and convincing analysis of the regulatory regions that control the expression of the entpd5a gene in each cell population. Determination of transcriptional activities in developing zebrafish, again combined with ATAC data and expression data of putative regulators, results in a compelling and detailed picture of the regulatory mechanisms governing the expression of this crucial gene.

Strengths:

The major strength of this paper is the clever combination of RNA-Seq and ATAC analysis, further combined with functional transcriptional analysis of the regulatory elements of one crucial gene. This results in a very compelling story.

Weaknesses:

No major weaknesses were identified, except for all the follow-up experiments that one can think of, but that would be outside of the scope of this paper.

**Reviewer #2 (Public Review):**

Summary:

Complementary to mammalian models, zebrafish has emerged as a powerful system to study vertebrate development and to serve as a go-to model for many human disorders. All vertebrates share the ancestral capacity to form a skeleton. Teleost fish models have been a key model to understand the foundations of skeletal development and plasticity, pairing with more classical work in amniotes such as the chicken and mouse. However, the genetic foundation of the diversity of skeletal programs in teleosts has been hampered by mapping similarities from amniotes back and not objectively establishing more ancestral states. This is most obvious in systematic, objective analysis of transcriptional regulation and tissue specification in differentiated skeletal tissues. Thus, the molecular events regulating bone-producing cells in teleosts have remained largely elusive. In this study, Petratou et al. leverage spatial experimental delineation of specific skeletal tissues -- that they term 'classical' vs 'non-classical' osteoblasts -- with associated cartilage of the endo/peri-chondrial skeleton and inter-segmental regions of the forming spine during development of the zebrafish, to delineate molecular specification of these cells by current chromatin and transcriptome analysis. The authors further show functional evidence of the utility of these datasets to identify functional enhancer regions delineating entp5 expression in 'classical' or 'non-classical' osteoblast populations. By integration with paired RNA-seq, they delineate broad patterns of transcriptional regulation of these populations as well as specific details of regional regulation via predictive binding sites within ATACseq profiles. Overall the paper was very well written and provides an essential contribution to the field that will provide a foundation to promote modeling of skeletal development and disease in an evolutionary and developmentally informed manner.

Strengths:

Taken together, this study provides a comprehensive resource of ATAC-seq and RNA-seq data that will be very useful for a wide variety of researchers studying skeletal development and bone pathologies. The authors show specificity in the different skeletal lineages and show the utility of the broad datasets for defining regulatory control of gene regulation in these different lineages, providing a foundation for hypothesis testing of not only agents of skeletal change in evolution but also function of genes and variations of unknown significance as it pertains to disease modeling in zebrafish. The paper is excellently written, integrating a complex history and experimental analysis into a useful and coherent whole. The terminology of 'classical' and 'non-classical' will be useful for the community in discussing the biology of skeletal lineages and their regulation.

Weaknesses:

Two items arose that were not critical weaknesses but areas for extending the description of methods and integration into the existing data on the role of non-classical osteoblasts and establishment/canalization of this lineage of skeletal cells.

(1) In reading the text it was unclear how specific the authors' experimental dissection of the head/trunk was in isolating different entp5a osteoblast populations. Obviously, this was successful given the specificity in DEG of results, however, analysis of contaminating cells/lineages in each population would be useful - e.g. using specific marker genes to assess. The text uses terms such as 'specific to' and 'enriched in' without seemingly grounded meaning of the accuracy of these comments. Is it really specific - e.g. not seen in one or other dataset - or is there some experimental variation in this?

We thank the reviewer for pointing this out. Given that the separation from head and trunk is done manually, there will be some experimental variability. We have used anatomical hallmarks (cleithrum and swim bladder), and therefore would expect the variability to be small. Regarding classical osteoblasts contaminating trunk tissue, head removal was consistently performed using the aforementioned anatomical hallmarks in a manner that ensures that the cleithrum does not remain in the trunk tissue. In order to alleviate concerns regarding trunk cell populations contaminating cranial populations, and to further clarify our strategy, we add the following statement to the Materials and Methods section: “The procedure does not allow for a complete separation of notochordal non-classical osteoblasts from cranial classical osteoblasts, as the notochord extends into the cranium. However, the amount of sheath cells in that portion of the notochord is negligible, compared both to the number of classical (cranial) osteoblasts in head samples, and to notochord cells isolated in trunk samples.”

(2) Further, it would be valuable to discuss NSC-specific genes such as calymmin (Peskin 2020) which has species and lineage-specific regulation of non-classical osteoblasts likely being a key mechanistic node for ratcheting centra-specific patterning of the spine in teleost fishes. What are dynamics observed in this gene in datasets between the different populations, especially when compared with paralogues - are there obvious cis-regulatory changes that correlate with the co-option of this gene in the early regulation of non-classical osteoblasts? The addition of this analysis/discussion would anchor discussions of the differential between different osteoblasts lineages in the paper.

This is an interesting concept and idea, that we will consider in a possible revision or, if requiring substantial additional efforts, in a possible new research line. An excellent starting point for further studies using our datasets.

**Reviewer #3 (Public Review):**

Summary:

This study characterizes classical and nonclassical osteoblasts as both types were analyzed independently (integrated ATAC-seq and RNAseq). It was found that gene expression in classical and nonclassical osteoblasts is not regulated in the same way. In classical osteoblasts, Dlx family factors seem to play an important role, while Hox family factors are involved in the regulation of spinal ossification by nonclassical osteoblasts. In the second part of the study, the authors focus on the promoter structure of entpd5a. Through the identification of enhancers, they reveal complex modes of regulation of the gene. The authors suggest candidate transcription factors that likely act on the identified enhancer elements. All the results taken together provide comprehensive new insights into the process of bone development, and point to spatio-temporally regulated promoter/enhancer interactions taking place at the entpd5a locus.

Strengths:

The authors have succeeded in justifying a sound and consistent buildup of their experiments, and meaningfully integrating the results into the design of each of their follow-up experiments. The data are solid, insightfully presented, and the conclusion valid. This makes this manuscript of great value and interest to those studying (fundamental) skeletal biology.

Weaknesses:

The study is solidly constructed, the manuscript is clearly written and the discussion is meaningful - I see no real weaknesses.

**Recommendations for the authors:**

**Reviewer #1 (Recommendations For The Authors):**

Minor issues that may need to be addressed or detailed:

Supplementary Figures 1I-J, text page 4, line 24: "photoconversion and imaging": this needs some more detailed description: green fluorescent cells should be actively expressing Kaede, but only if there is a delay between photoconversion and imaging. What is the reason that Supplementary Figure 1F shows mainly green fluorescent cells, contrary to 1G-J?

In our experiments, we could see new Kaede expression under the control of the *entpd5a* promoter region within 1.5 hours of photoconversion, as shown in Suppl. Figure 1E-H, suggesting that this time window was sufficient for protein generation. The reason for Suppl. Fig 1F showing more green fluorescence we believe relates to the high rate of transcriptional activity at that stage, in the entirety of the notochord progenitor cells. In addition, this is an effect which we attribute to the relatively small number of cells producing red fluorescence at that stage, due to photoconversion of only a few Kaede+ cells at the 15 somites stage (Suppl. Fig. 1E). Therefore, the masking effect of the green fluorescence by the red is not as significant as in G and H, where the red fluorescence resulting from photoconversion right after imaging at 18s and 21s, respectively, significantly overlaps with new green fluorescence. This can be seen in the image as the presence of orange fluorescence in G and H, instead of the clear red shown in E, I and J.

In addition to this, we would like to point out that in Suppl. Fig. 1I, J the reason that green fluorescence is only detected in the ventral region of the notochord, is because the promoter of *entpd5a* only remains active in the ventral-most sheath cells at that stage. This is stated in the results section of the main text, first subsection, paragraph 3. The reason for this very interesting, strictly localised expression pattern remains unclear.

Somewhat intriguing: green fluorescence in Figure 1B, C (osx:GAL4FF) and Supplementary Figure 1C (entpd5a:GAL4FF) in the CNS? Would that be an artefact of the GAL4FF/UAS:GFP system?

We are confident that the fluorescence pointed out by the reviewer is not an artefact of the GAL4FF/UAS system, for a few reasons. Firstly, *osx* (Sp7) has been shown to be expressed and to function in the nervous system in mice (Park et al, BBRC, 2011; Elbaz et al, Neuron, 2023). Secondly, not only *osx*, but also *entpd5a* can be readily detected in a subset of cranial and spinal neurons in early development using the *entpd5a:GAL4FF; UAS:GFP* transgenic line (Suppl. Fig 1C). Finally, when establishing transgenic lines with the *entpd5a(1.1):GFP* construct, expression was almost invariably present in diverse elements of the nervous system, but not in bone (data not shown). This led us to hypothesise that the minimal promoter of *entpd5a* (and possibly also that of *osx*) is activated by transcription factors active in the nervous system, and this effect is likely controlled by the surrounding enhancers, but also the genome location. It is unclear at present what the endogenous neural expression of the two genes is like, and we did not further investigate this in this study, as the focus was on the skeleton.

Figure 2: What exactly is "Corrected Total Cell Fluorescence"? Is it green + red fluorescence?

We thank the reviewer for pointing out the absence of more information on this. Corrected total cell fluorescence does not correspond to green+ red fluorescence, rather it is calculated as follows for a single channel:

CTCF = Integrated Density – (Area of selected cell X Mean fluorescence of background readings)

More details can be found in the following website: https://theolb.readthedocs.io/en/latest/imaging/measuring-cell-fluorescence-using-imagej.html

We have edited the Materials and Methods section under “Imaging and image analysis” to include the aforementioned information.

Page 11, line 34: The authors may have missed the recently published "Raman et al., Biomolecules 2024 Vol. 14; doi:10.3390/biom14020139" describing RNA-Seq in 4 dpf osterix+ osteoblasts.

We thank the reviewer for drawing our attention to the Raman et al publication. The reference has now been added in the manuscript.

Figure 5A and B: use a higher resolution version to make the numbers and gene names more readable. Figures 5C and 6A could also use a larger font for the text and numbers.

High resolution files are now included with the revised manuscript, which should significantly help in making figures more easily readable. Although we agree with the reviewer that larger fonts would improve readability, due to the nature of the graphs (very small spaces in some cases, where the numbers would have to fit) this would not be easy to achieve. However, we believe that this issue will be resolved with the availability of higher resolution files. If readability remains a concern, we would be happy to attempt re-organising the graphs to allow for larger fonts.

**Reviewer #2 (Recommendations For The Authors):**

I suggest no further experiments, but do suggest that a few points be clarified.

In the Discussion, the text "the less evolved osteoblasts of fish and amphibians..." is not accurate. These cells are not less evolved as they represent an independent lineage to tetrapods that have evolved with different stresses for a similar time. However, as teleost fishes and amphibians share characteristics and all share a common ancestor, these signatures represent a putative ancestral state of skeletal differentiation not seen in amniotes, including humans.

We thank the reviewer for pointing out the unfortunate phrasing. The text has now been modified as follows: “Specifically, the osteoblasts of teleost fish and amphibians, whose characteristics are putatively closer to a more ancestral state of skeletal differentiation compared to amniotes, appear to share gene expression with chondrocytes”.

The title could potentially be shortened to reach a broader audience by removing the initial clause of 'integration of ATAC and RNA seq' as this is a commonly performed analysis - "Chromatin and transcriptomic signature in classical and non-classical zebrafish osteoblasts indicate mechanisms of ancestral skeletal differentiation" is more descriptive of the findings and not focused on the method.

We have discussed this internally, but would prefer to retain the current title. The reason is (1) because we would like to see our methodology and datasets be used as platform for further studies, and the current title, in our opinion, facilitates this. In regards to replacing “mechanisms of *entpd5a* regulation” with “mechanisms of ancestral skeletal differentiation”, we think this does not give an accurate description of our work, which is primarily focused on elucidating *entpd5a* promoter dynamics.

All datasets should be made available as soon as possible for use in the field.

The datasets (raw and processed) are available on the GEO database. The corresponding accession numbers can be found in our data availability statement.

Minor comments:

(1) Figure 1A. The labels are missing for grey and light blue structures.

These structures are together making up the “notochord sheath”, which is comprised of the basal lamina (grey), the medial layer of fibrillar collagen (light blue) and the outer layer of loosely arranged matrix (lighter blue). We modified the figure legend to indicate that the three layers all correspond to the notochord sheath.

(2) Figure 2A. The constructs in the lower part of the panel are not discussed in the legend and seem out of place in terms of data type and analysis.

We would argue that indicating which non-coding regions and which ATAC peaks were responsible for driving GFP expression in each construct aids in a better understanding of our results. We thank the reviewer for pointing out the lack of mention of these constructs in the figure legend. This issue has now been resolved.

(3) Be wary of red/green color combinations, especially in the figures where these are juxtaposed with each other.

We apologise for the use of red/green colour. Although it is not possible for this manuscript to change the colour patterns, we will make sure to avoid the use of these colours in conjunction in the future.

(4) The use of fish as a term should be classified as teleost fish, as authors are not addressing non-teleost basal ray-finned fishes or the fact that tetrapods are within bony fishes overall.

This is well spotted, we have now remedied this by editing the manuscript. Where the term “fish” was used, we now state “teleost fish”.

(5) Age information is missing in several Figures (e.g. 1D and 2C).

In some of the figures space constrains did not allow for including the stage on the figure itself. However, we have made sure that in those cases the stage is incorporated in the figure legend.

(6) The resolution of several Figures (e.g. Figure 5 and Supplementary Figure 3) is low.

We address this issue by providing high resolution figures with the revised manuscript.

(7) In the sentence (top page before Discussion) "The same conclusion was reached upon isolation from these three..", it was unclear what 'upon isolation' referred to.

We agree with the reviewer that this phrasing is unclear. To enhance clarity, the manuscript now reads as follows: “The same conclusion was reached upon isolation of the DEGs highlighted by our RNA-seq results, from the three aforementioned groups of genes associated with ATAC peaks for each cell population.”
